# Crystal structure of chlorfluazuron

**DOI:** 10.1107/S2056989014026632

**Published:** 2015-01-01

**Authors:** Seonghwa Cho, Jineun Kim, Sangjin Lee, Tae Ho Kim

**Affiliations:** a Department of Chemistry and Research Institute of Natural Sciences, Gyeongsang National University, Jinju 660-701, Republic of Korea

**Keywords:** crystal structure, chlorfluazuron, urea, insecticidal properties, Cl⋯Cl contacts

## Abstract

The title compound (systematic name: 1-{3,5-di­chloro-4-[3-chloro-5-(tri­fluoro­meth­yl)pyridin-2-yl­oxy]phen­yl}-3-(2,6-difluoro­benzo­yl)urea), C_20_H_9_Cl_3_F_5_N_3_O_3_, is a benzoyl­phenyl­urea insecticide. The dihedral angles between the planes of the central di­chloro­phenyl and the terminal di­fluoro­phenyl and chloro­pyridyl rings are 79.51 (6) and 78.84 6)°, respectively. In the crystal, pairs of N—H⋯O hydrogen bonds link adjacent mol­ecules, forming *R*_2_^2^(8) inversion dimers. In addition, the dimers are linked by short F⋯Cl [3.1060 (16) Å] and Cl⋯Cl [3.2837 (7) Å] contacts, as well as weak inter­molecular π–π inter­actions [ring centroid separation = 3.6100 (11) and 3.7764 (13) Å], resulting in a two-dimensional architecture parallel to (111).

## Related literature   

For information on the insecticidal properties of the title compound, see: Choi *et al.* (2011 ▸); Lee *et al.* (2013 ▸). For a related crystal structure, see: Jeon *et al.* (2014 ▸).
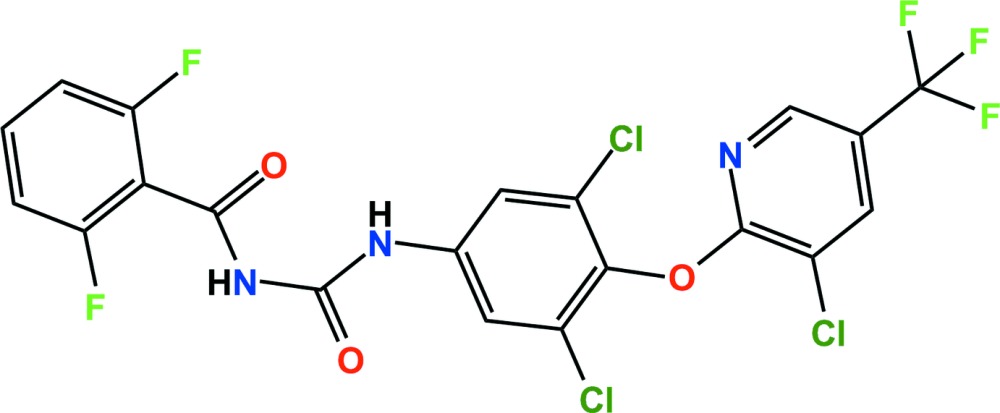


## Experimental   

### Crystal data  

C_20_H_9_Cl_3_F_5_N_3_O_3_
*M* *_r_* = 540.65
Triclinic, 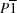

*a* = 8.5805 (3) Å
*b* = 10.1281 (4) Å
*c* = 12.5883 (4) Å
α = 79.498 (2)°
β = 82.930 (2)°
γ = 83.485 (2)°
*V* = 1062.82 (7) Å^3^
*Z* = 2
Mo *K*α radiation
μ = 0.51 mm^−1^
*T* = 173 K
0.28 × 0.12 × 0.05 mm

### Data collection  

Bruker APEXII CCD diffractometer
Absorption correction: multi-scan (*SADABS*; Bruker, 2009 ▸) *T*_min_ = 0.872, *T*_max_ = 0.975
19710 measured reflections
5259 independent reflections
4090 reflections with *I* > 2σ(*I*)
*R*_int_ = 0.040

### Refinement  

*R*[*F* ^2^ > 2σ(*F* ^2^)] = 0.046
*wR*(*F* ^2^) = 0.127
*S* = 1.06
5259 reflections
307 parameters
H-atom parameters constrained
Δρ_max_ = 0.58 e Å^−3^
Δρ_min_ = −0.54 e Å^−3^

### 

Data collection: *APEX2* (Bruker, 2009 ▸); cell refinement: *SAINT* (Bruker, 2009 ▸); data reduction: *SAINT*; program(s) used to solve structure: *SHELXTL* (Sheldrick, 2008 ▸); program(s) used to refine structure: *SHELXTL*; molecular graphics: *DIAMOND* (Brandenburg, 2010 ▸); software used to prepare material for publication: *SHELXTL*.

## Supplementary Material

Crystal structure: contains datablock(s) global, I. DOI: 10.1107/S2056989014026632/sj5431sup1.cif

Structure factors: contains datablock(s) I. DOI: 10.1107/S2056989014026632/sj5431Isup2.hkl

Supporting information file. DOI: 10.1107/S2056989014026632/sj5431Isup3.cml

**Fig. 1.**
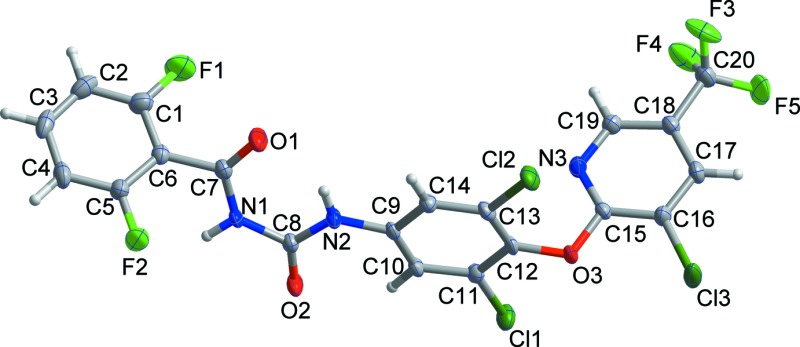
The asymmetric unit of the title compound with the atom numbering scheme. Displacement ellipsoids are drawn at the 50% probability level. H atoms are shown as small spheres of arbitrary radius.

**Fig. 2.**
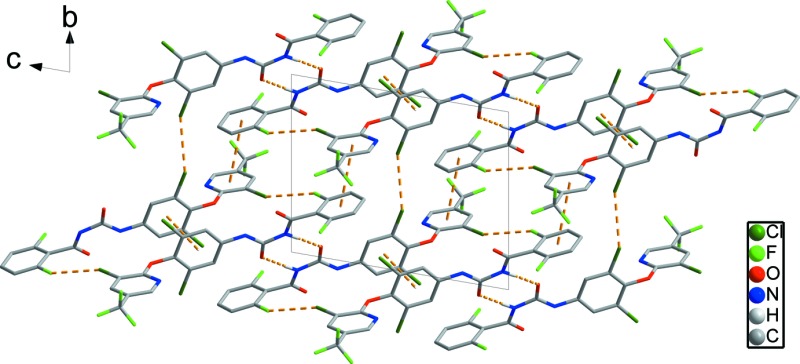
Crystal packing viewed along the a axis. The intermolecular N—H···O hydrogen bonds, short F···Cl, Cl···Cl contacts and π–π interactions are shown as dashed lines.

. DOI: 10.1107/S2056989014026632/sj5431fig1.tif
The asymmetric unit of the title compound with the atom numbering scheme. Displacement ellipsoids are drawn at the 50% probability level. H atoms are shown as small spheres of arbitrary radius.

a . DOI: 10.1107/S2056989014026632/sj5431fig2.tif
Crystal packing viewed along the *a* axis. The inter­molecular N—H⋯O hydrogen bonds, short F⋯Cl, Cl⋯Cl contacts and π–π inter­actions are shown as dashed lines.

CCDC reference: 1037499

Additional supporting information:  crystallographic information; 3D view; checkCIF report

## Figures and Tables

**Table 1:** Hydrogen-bond geometry (, )

*D*H*A*	*D*H	H*A*	*D* *A*	*D*H*A*
N1H1O2^i^	0.88	1.96	2.837(2)	175

Symmetry code: (i) 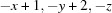.

## supplementary crystallographic information

### S1. Comment

Chlorfluazuron, C_20_H_9_Cl_3_F_5_N_3_O_3_, is a benzoylphenylurea which has been recognized as one of the promising insecticides with great potential for use in controlling insect attack on a broad range of fruits and vegetables (Lee *et al.*, 2013; Choi *et al.*, 2011). Its crystal structure is reported herein. In this compound (Scheme 1, Fig. 1), the dihedral angles between the central dichlorophenyl and the terminal difluorophenyland chloropyridyl rings are 79.51 (6) and 78.84 (6)°, respectively. All bond lengths and bond angles are normal and comparable to those observed in the crystal structure of a similar compound (Jeon *et al.*, 2014).

The crystal structure, (Fig. 2), is stabilized by N—H···O hydrogen bonds (Table 1), forming *R*_2_^2^(8) inversion dimers. In addition, the dimers are linked by short F2···Cl3^ii^ [3.1060 (16) Å] and Cl1···Cl1^iii^ [3.2837 (7) Å] contacts as well as two weak intermolecular offset π–π stacking interactions [*Cg*1···*Cg*3^iv^ = 3.7764 (13)Å. *Cg*1 and *Cg*3 are the centroids of the C1—C2—C3—C4—C5—C6 and C15—C16—C17—C18—C19—N3 rings, respectively. *Cg*2···*Cg*2^ii^ = 3.6100 (11) Å. *Cg*2 is the centroid of the C9—C10—C11—C12—C13—C14 ring. (Symmetry codes: (ii), -*x* + 1, -*y* + 2, -*z* + 1; (iii) -*x* + 1, -*y* + 1, -*z* + 1 and (iv) *x*, *y* + 1, *z* - 1)], resulting in a two-dimensional architecture parallel to the (111) plane.

### S2. Experimental

The title compound was purchased from the Dr. Ehrenstorfer GmbH Company. Slow evaporation of a solution in CHCl_3_ gave single crystals suitable for X-ray analysis.

### S3. Refinement

All H-atoms were positioned geometrically and refined using a riding model with d(C—H) = 0.95 Å, *U*_iso_ = 1.2*U*_eq_(C) for aromatic C—H and d(N—H) = 0.88 Å, *U*_iso_ = 1.2*U*_eq_(C) for urea group.

### Crystal data

**Table:**

C_20_H_9_Cl_3_F_5_N_3_O_3_	*Z* = 2
*M**_r_* = 540.65	*F*(000) = 540
Triclinic, *P*1	*D*_x_ = 1.689 Mg m^−^^3^
Hall symbol: -P 1	Mo *K*α radiation, λ = 0.71073 Å
*a* = 8.5805 (3) Å	Cell parameters from 6417 reflections
*b* = 10.1281 (4) Å	θ = 2.4–28.1°
*c* = 12.5883 (4) Å	µ = 0.51 mm^−^^1^
α = 79.498 (2)°	*T* = 173 K
β = 82.930 (2)°	Plate, colourless
γ = 83.485 (2)°	0.28 × 0.12 × 0.05 mm
*V* = 1062.82 (7) Å^3^	

### Data collection

**Table:**

Bruker APEXII CCD diffractometer	5259 independent reflections
Radiation source: fine-focus sealed tube	4090 reflections with *I* > 2σ(*I*)
Graphite monochromator	*R*_int_ = 0.040
φ and ω scans	θ_max_ = 28.3°, θ_min_ = 1.7°
Absorption correction: multi-scan (*SADABS*; Bruker, 2009)	*h* = −11→10
*T*_min_ = 0.872, *T*_max_ = 0.975	*k* = −13→13
19710 measured reflections	*l* = −16→16

### Refinement

**Table:**

Refinement on *F*^2^	Primary atom site location: structure-invariant direct methods
Least-squares matrix: full	Secondary atom site location: difference Fourier map
*R*[*F*^2^ > 2σ(*F*^2^)] = 0.046	Hydrogen site location: inferred from neighbouring sites
*wR*(*F*^2^) = 0.127	H-atom parameters constrained
*S* = 1.06	*w* = 1/[σ^2^(*F*_o_^2^) + (0.0631*P*)^2^ + 0.4257*P*] where *P* = (*F*_o_^2^ + 2*F*_c_^2^)/3
5259 reflections	(Δ/σ)_max_ = 0.001
307 parameters	Δρ_max_ = 0.58 e Å^−^^3^
0 restraints	Δρ_min_ = −0.54 e Å^−^^3^

### Special details

**Table:**

Geometry. All e.s.d.'s (except the e.s.d. in the dihedral angle between two l.s. planes) are estimated using the full covariance matrix. The cell e.s.d.'s are taken into account individually in the estimation of e.s.d.'s in distances, angles and torsion angles; correlations between e.s.d.'s in cell parameters are only used when they are defined by crystal symmetry. An approximate (isotropic) treatment of cell e.s.d.'s is used for estimating e.s.d.'s involving l.s. planes.
Refinement. Refinement of *F*^2^ against ALL reflections. The weighted *R*-factor *wR* and goodness of fit *S* are based on *F*^2^, conventional *R*-factors *R* are based on *F*, with *F* set to zero for negative *F*^2^. The threshold expression of *F*^2^ > σ(*F*^2^) is used only for calculating *R*-factors(gt) *etc*. and is not relevant to the choice of reflections for refinement. *R*-factors based on *F*^2^ are statistically about twice as large as those based on *F*, and *R*- factors based on ALL data will be even larger.

### Fractional atomic coordinates and isotropic or equivalent isotropic displacement parameters (Å^2^)

**Table:**

	*x*	*y*	*z*	*U*_iso_*/*U*_eq_	
Cl1	0.51049 (7)	0.65788 (5)	0.51055 (4)	0.03275 (16)	
Cl2	0.82607 (7)	1.05194 (6)	0.59008 (4)	0.03172 (15)	
Cl3	0.64381 (7)	0.71391 (8)	0.88086 (5)	0.0459 (2)	
F1	1.05653 (18)	1.14118 (18)	−0.13423 (14)	0.0590 (5)	
F2	0.54499 (16)	1.35343 (16)	−0.10772 (12)	0.0470 (4)	
F3	1.33225 (17)	0.60959 (17)	0.78423 (15)	0.0549 (5)	
F4	1.28741 (18)	0.43892 (17)	0.72279 (14)	0.0552 (5)	
F5	1.2067 (2)	0.4572 (2)	0.88756 (14)	0.0753 (7)	
O1	0.86100 (19)	1.20897 (17)	0.06450 (12)	0.0347 (4)	
O2	0.51802 (19)	0.94182 (16)	0.13689 (12)	0.0323 (4)	
O3	0.66744 (16)	0.79859 (14)	0.64590 (11)	0.0237 (3)	
N1	0.6634 (2)	1.09625 (17)	0.02702 (13)	0.0229 (4)	
H1	0.6120	1.0859	−0.0268	0.027*	
N2	0.6858 (2)	1.04878 (17)	0.21206 (13)	0.0218 (4)	
H2	0.7469	1.1153	0.1963	0.026*	
N3	0.9237 (2)	0.70890 (17)	0.60728 (14)	0.0245 (4)	
C1	0.9398 (3)	1.2235 (2)	−0.1811 (2)	0.0323 (5)	
C2	0.9644 (3)	1.2813 (3)	−0.2889 (2)	0.0382 (6)	
H2A	1.0617	1.2633	−0.3309	0.046*	
C3	0.8436 (3)	1.3662 (3)	−0.33402 (18)	0.0382 (6)	
H3	0.8589	1.4080	−0.4080	0.046*	
C4	0.7021 (3)	1.3915 (2)	−0.27473 (18)	0.0356 (6)	
H4	0.6197	1.4502	−0.3066	0.043*	
C5	0.6822 (3)	1.3296 (2)	−0.16762 (18)	0.0275 (5)	
C6	0.7993 (2)	1.2441 (2)	−0.11733 (16)	0.0220 (4)	
C7	0.7787 (2)	1.1828 (2)	0.00044 (16)	0.0228 (4)	
C8	0.6163 (2)	1.0217 (2)	0.12916 (16)	0.0222 (4)	
C9	0.6721 (2)	0.98326 (19)	0.32049 (15)	0.0190 (4)	
C10	0.5976 (2)	0.86569 (19)	0.35643 (16)	0.0207 (4)	
H10	0.5464	0.8274	0.3081	0.025*	
C11	0.5998 (2)	0.80569 (19)	0.46473 (16)	0.0204 (4)	
C12	0.6729 (2)	0.85890 (19)	0.53728 (15)	0.0202 (4)	
C13	0.7419 (2)	0.9786 (2)	0.50062 (16)	0.0221 (4)	
C14	0.7433 (2)	1.0399 (2)	0.39305 (16)	0.0221 (4)	
H14	0.7929	1.1208	0.3685	0.026*	
C15	0.8040 (2)	0.73051 (19)	0.67978 (16)	0.0192 (4)	
C16	0.8070 (2)	0.6866 (2)	0.79131 (16)	0.0237 (4)	
C17	0.9430 (2)	0.6189 (2)	0.82825 (17)	0.0258 (5)	
H17	0.9492	0.5881	0.9037	0.031*	
C18	1.0710 (2)	0.5965 (2)	0.75305 (17)	0.0235 (4)	
C19	1.0565 (3)	0.6423 (2)	0.64440 (17)	0.0250 (4)	
H19	1.1445	0.6262	0.5932	0.030*	
C20	1.2233 (3)	0.5247 (2)	0.78821 (19)	0.0321 (5)	

### Atomic displacement parameters (Å^2^)

**Table:**

	*U*^11^	*U*^22^	*U*^33^	*U*^12^	*U*^13^	*U*^23^
Cl1	0.0441 (3)	0.0284 (3)	0.0261 (3)	−0.0152 (2)	−0.0103 (2)	0.0063 (2)
Cl2	0.0376 (3)	0.0384 (3)	0.0237 (3)	−0.0096 (2)	−0.0131 (2)	−0.0067 (2)
Cl3	0.0296 (3)	0.0784 (5)	0.0193 (3)	0.0167 (3)	0.0006 (2)	0.0036 (3)
F1	0.0343 (8)	0.0660 (11)	0.0626 (11)	0.0190 (8)	0.0036 (8)	0.0059 (9)
F2	0.0289 (7)	0.0586 (9)	0.0402 (8)	0.0124 (7)	0.0018 (6)	0.0122 (7)
F3	0.0285 (8)	0.0604 (10)	0.0824 (13)	−0.0001 (7)	−0.0263 (8)	−0.0180 (9)
F4	0.0402 (9)	0.0576 (10)	0.0721 (12)	0.0233 (8)	−0.0194 (8)	−0.0305 (9)
F5	0.0415 (9)	0.1138 (16)	0.0434 (10)	0.0300 (10)	−0.0047 (8)	0.0351 (10)
O1	0.0393 (9)	0.0434 (9)	0.0243 (8)	−0.0195 (8)	−0.0120 (7)	0.0024 (7)
O2	0.0392 (9)	0.0394 (9)	0.0200 (8)	−0.0185 (7)	−0.0128 (7)	0.0059 (6)
O3	0.0189 (7)	0.0334 (8)	0.0160 (7)	0.0013 (6)	−0.0054 (6)	0.0030 (6)
N1	0.0234 (9)	0.0305 (9)	0.0154 (8)	−0.0070 (7)	−0.0089 (7)	0.0019 (7)
N2	0.0266 (9)	0.0222 (8)	0.0167 (8)	−0.0069 (7)	−0.0071 (7)	0.0022 (6)
N3	0.0261 (9)	0.0269 (9)	0.0188 (9)	0.0010 (7)	−0.0039 (7)	−0.0006 (7)
C1	0.0271 (12)	0.0321 (12)	0.0354 (13)	−0.0008 (9)	0.0004 (10)	−0.0039 (10)
C2	0.0398 (14)	0.0443 (14)	0.0311 (13)	−0.0145 (11)	0.0123 (11)	−0.0124 (11)
C3	0.0571 (16)	0.0417 (14)	0.0184 (11)	−0.0247 (12)	−0.0026 (11)	−0.0002 (10)
C4	0.0447 (14)	0.0369 (13)	0.0243 (12)	−0.0093 (11)	−0.0131 (11)	0.0071 (10)
C5	0.0247 (11)	0.0326 (11)	0.0233 (11)	−0.0045 (9)	−0.0030 (9)	0.0016 (9)
C6	0.0250 (11)	0.0219 (10)	0.0196 (10)	−0.0056 (8)	−0.0039 (8)	−0.0017 (8)
C7	0.0223 (10)	0.0246 (10)	0.0208 (10)	−0.0009 (8)	−0.0049 (8)	−0.0011 (8)
C8	0.0226 (10)	0.0248 (10)	0.0185 (10)	−0.0024 (8)	−0.0058 (8)	0.0006 (8)
C9	0.0181 (9)	0.0221 (9)	0.0159 (9)	0.0020 (7)	−0.0060 (7)	−0.0007 (7)
C10	0.0226 (10)	0.0225 (10)	0.0176 (10)	−0.0019 (8)	−0.0085 (8)	−0.0013 (8)
C11	0.0214 (10)	0.0177 (9)	0.0210 (10)	−0.0013 (7)	−0.0043 (8)	0.0008 (7)
C12	0.0202 (10)	0.0246 (10)	0.0142 (9)	0.0028 (8)	−0.0068 (8)	0.0015 (8)
C13	0.0219 (10)	0.0266 (10)	0.0200 (10)	−0.0006 (8)	−0.0090 (8)	−0.0063 (8)
C14	0.0229 (10)	0.0236 (10)	0.0201 (10)	−0.0052 (8)	−0.0056 (8)	−0.0009 (8)
C15	0.0180 (9)	0.0213 (9)	0.0185 (10)	−0.0019 (7)	−0.0065 (8)	−0.0007 (7)
C16	0.0231 (10)	0.0298 (11)	0.0168 (10)	0.0009 (8)	−0.0020 (8)	−0.0021 (8)
C17	0.0272 (11)	0.0321 (11)	0.0178 (10)	−0.0016 (9)	−0.0079 (9)	−0.0004 (8)
C18	0.0218 (10)	0.0251 (10)	0.0233 (10)	−0.0007 (8)	−0.0067 (8)	−0.0015 (8)
C19	0.0230 (10)	0.0295 (11)	0.0216 (10)	−0.0006 (8)	−0.0018 (8)	−0.0035 (8)
C20	0.0264 (11)	0.0389 (13)	0.0287 (12)	0.0045 (10)	−0.0069 (9)	−0.0020 (10)

### Geometric parameters (Å, º)

**Table:**

Cl1—C11	1.7315 (19)	C2—H2A	0.9500
Cl2—C13	1.723 (2)	C3—C4	1.368 (4)
Cl3—C16	1.716 (2)	C3—H3	0.9500
F1—C1	1.350 (3)	C4—C5	1.378 (3)
F2—C5	1.340 (3)	C4—H4	0.9500
F3—C20	1.331 (3)	C5—C6	1.385 (3)
F4—C20	1.333 (3)	C6—C7	1.497 (3)
F5—C20	1.311 (3)	C9—C10	1.392 (3)
O1—C7	1.217 (2)	C9—C14	1.394 (3)
O2—C8	1.217 (2)	C10—C11	1.389 (3)
O3—C15	1.365 (2)	C10—H10	0.9500
O3—C12	1.390 (2)	C11—C12	1.384 (3)
N1—C7	1.366 (3)	C12—C13	1.387 (3)
N1—C8	1.405 (2)	C13—C14	1.382 (3)
N1—H1	0.8800	C14—H14	0.9500
N2—C8	1.347 (3)	C15—C16	1.396 (3)
N2—C9	1.402 (2)	C16—C17	1.373 (3)
N2—H2	0.8800	C17—C18	1.386 (3)
N3—C15	1.314 (3)	C17—H17	0.9500
N3—C19	1.347 (3)	C18—C19	1.378 (3)
C1—C2	1.377 (3)	C18—C20	1.497 (3)
C1—C6	1.381 (3)	C19—H19	0.9500
C2—C3	1.379 (4)		
			
C15—O3—C12	116.53 (15)	C11—C10—H10	120.8
C7—N1—C8	128.40 (17)	C9—C10—H10	120.8
C7—N1—H1	115.8	C12—C11—C10	122.32 (18)
C8—N1—H1	115.8	C12—C11—Cl1	118.64 (15)
C8—N2—C9	127.54 (17)	C10—C11—Cl1	119.03 (15)
C8—N2—H2	116.2	C11—C12—C13	118.40 (17)
C9—N2—H2	116.2	C11—C12—O3	120.52 (17)
C15—N3—C19	117.22 (17)	C13—C12—O3	120.94 (17)
F1—C1—C2	119.5 (2)	C14—C13—C12	120.64 (18)
F1—C1—C6	117.4 (2)	C14—C13—Cl2	119.64 (15)
C2—C1—C6	123.2 (2)	C12—C13—Cl2	119.72 (15)
C1—C2—C3	118.0 (2)	C13—C14—C9	120.16 (18)
C1—C2—H2A	121.0	C13—C14—H14	119.9
C3—C2—H2A	121.0	C9—C14—H14	119.9
C4—C3—C2	121.5 (2)	N3—C15—O3	119.22 (17)
C4—C3—H3	119.2	N3—C15—C16	123.69 (18)
C2—C3—H3	119.2	O3—C15—C16	117.09 (18)
C3—C4—C5	118.4 (2)	C17—C16—C15	118.60 (19)
C3—C4—H4	120.8	C17—C16—Cl3	120.33 (16)
C5—C4—H4	120.8	C15—C16—Cl3	121.07 (16)
F2—C5—C4	119.4 (2)	C16—C17—C18	118.51 (19)
F2—C5—C6	117.64 (18)	C16—C17—H17	120.7
C4—C5—C6	122.9 (2)	C18—C17—H17	120.7
C1—C6—C5	116.01 (19)	C19—C18—C17	118.80 (19)
C1—C6—C7	121.58 (19)	C19—C18—C20	120.08 (19)
C5—C6—C7	122.35 (19)	C17—C18—C20	121.11 (19)
O1—C7—N1	124.57 (19)	N3—C19—C18	123.18 (19)
O1—C7—C6	121.15 (18)	N3—C19—H19	118.4
N1—C7—C6	114.27 (17)	C18—C19—H19	118.4
O2—C8—N2	125.62 (18)	F5—C20—F3	107.5 (2)
O2—C8—N1	119.61 (18)	F5—C20—F4	108.0 (2)
N2—C8—N1	114.76 (17)	F3—C20—F4	104.67 (19)
C10—C9—C14	120.10 (18)	F5—C20—C18	112.37 (19)
C10—C9—N2	123.68 (18)	F3—C20—C18	111.92 (19)
C14—C9—N2	116.18 (17)	F4—C20—C18	112.02 (19)
C11—C10—C9	118.32 (18)		
			
F1—C1—C2—C3	−179.2 (2)	Cl1—C11—C12—O3	−3.3 (3)
C6—C1—C2—C3	1.5 (4)	C15—O3—C12—C11	107.5 (2)
C1—C2—C3—C4	−0.9 (4)	C15—O3—C12—C13	−77.0 (2)
C2—C3—C4—C5	−0.1 (4)	C11—C12—C13—C14	−2.7 (3)
C3—C4—C5—F2	179.6 (2)	O3—C12—C13—C14	−178.30 (18)
C3—C4—C5—C6	0.6 (4)	C11—C12—C13—Cl2	177.03 (16)
F1—C1—C6—C5	179.7 (2)	O3—C12—C13—Cl2	1.5 (3)
C2—C1—C6—C5	−1.0 (3)	C12—C13—C14—C9	1.4 (3)
F1—C1—C6—C7	2.6 (3)	Cl2—C13—C14—C9	−178.39 (15)
C2—C1—C6—C7	−178.1 (2)	C10—C9—C14—C13	0.8 (3)
F2—C5—C6—C1	−179.1 (2)	N2—C9—C14—C13	−177.08 (18)
C4—C5—C6—C1	−0.1 (3)	C19—N3—C15—O3	179.44 (17)
F2—C5—C6—C7	−2.0 (3)	C19—N3—C15—C16	−1.0 (3)
C4—C5—C6—C7	177.0 (2)	C12—O3—C15—N3	−9.7 (3)
C8—N1—C7—O1	0.0 (4)	C12—O3—C15—C16	170.78 (18)
C8—N1—C7—C6	179.18 (19)	N3—C15—C16—C17	1.1 (3)
C1—C6—C7—O1	60.5 (3)	O3—C15—C16—C17	−179.40 (18)
C5—C6—C7—O1	−116.5 (2)	N3—C15—C16—Cl3	−177.88 (16)
C1—C6—C7—N1	−118.8 (2)	O3—C15—C16—Cl3	1.6 (3)
C5—C6—C7—N1	64.3 (3)	C15—C16—C17—C18	−0.4 (3)
C9—N2—C8—O2	6.7 (4)	Cl3—C16—C17—C18	178.62 (16)
C9—N2—C8—N1	−174.42 (18)	C16—C17—C18—C19	−0.3 (3)
C7—N1—C8—O2	−175.0 (2)	C16—C17—C18—C20	179.3 (2)
C7—N1—C8—N2	6.1 (3)	C15—N3—C19—C18	0.3 (3)
C8—N2—C9—C10	8.7 (3)	C17—C18—C19—N3	0.3 (3)
C8—N2—C9—C14	−173.53 (19)	C20—C18—C19—N3	−179.2 (2)
C14—C9—C10—C11	−1.6 (3)	C19—C18—C20—F5	−163.0 (2)
N2—C9—C10—C11	176.18 (18)	C17—C18—C20—F5	17.4 (3)
C9—C10—C11—C12	0.1 (3)	C19—C18—C20—F3	76.0 (3)
C9—C10—C11—Cl1	−178.97 (15)	C17—C18—C20—F3	−103.6 (2)
C10—C11—C12—C13	2.0 (3)	C19—C18—C20—F4	−41.2 (3)
Cl1—C11—C12—C13	−178.89 (16)	C17—C18—C20—F4	139.2 (2)
C10—C11—C12—O3	177.58 (18)		

### Hydrogen-bond geometry (Å, º)

**Table:**

*D*—H···*A*	*D*—H	H···*A*	*D*···*A*	*D*—H···*A*
N1—H1···O2^i^	0.88	1.96	2.837 (2)	175

Symmetry code: (i) −*x*+1, −*y*+2, −*z*.

## References

[bb1] Brandenburg, K. (2010). *DIAMOND*. Crystal Impact GbR, Bonn, Germany.

[bb2] Bruker (2009). *APEX2*, *SAINT* and *SADABS*. Bruker AXS Inc., Madison, Wisconsin, USA.

[bb3] Choi, J. H., Mamun, M. I. R., Park, J. H., Shin, E. H. & Shim, J. H. (2011). *Bull. Environ. Contam. Toxicol.* **86**, 331–335. 10.1007/s00128-011-0191-5 21327612

[bb4] Jeon, Y., Kang, G., Lee, S. & Kim, T. H. (2014). *Acta Cryst.* E**70**, o1110. 10.1107/S1600536814020649 PMC4257195 25484700

[bb5] Lee, C. C., Man, C. N., Noor, N. M., Lajis, R. & Lee, C. Y. (2013). *J. Pestic. Sci.* **38**, 208–213.

[bb6] Sheldrick, G. M. (2008). *Acta Cryst.* A**64**, 112–122. 10.1107/S0108767307043930 18156677

