# Supplementary figures and images for: Crystal structure of chlorfluazuron
Source: Acta Crystallogr E Crystallogr Commun. 2015 Jan 1;71(Pt 1):o55. doi: 10.1107/S2056989014026632 (PMC4331875; doi:10.1107/S2056989014026632)

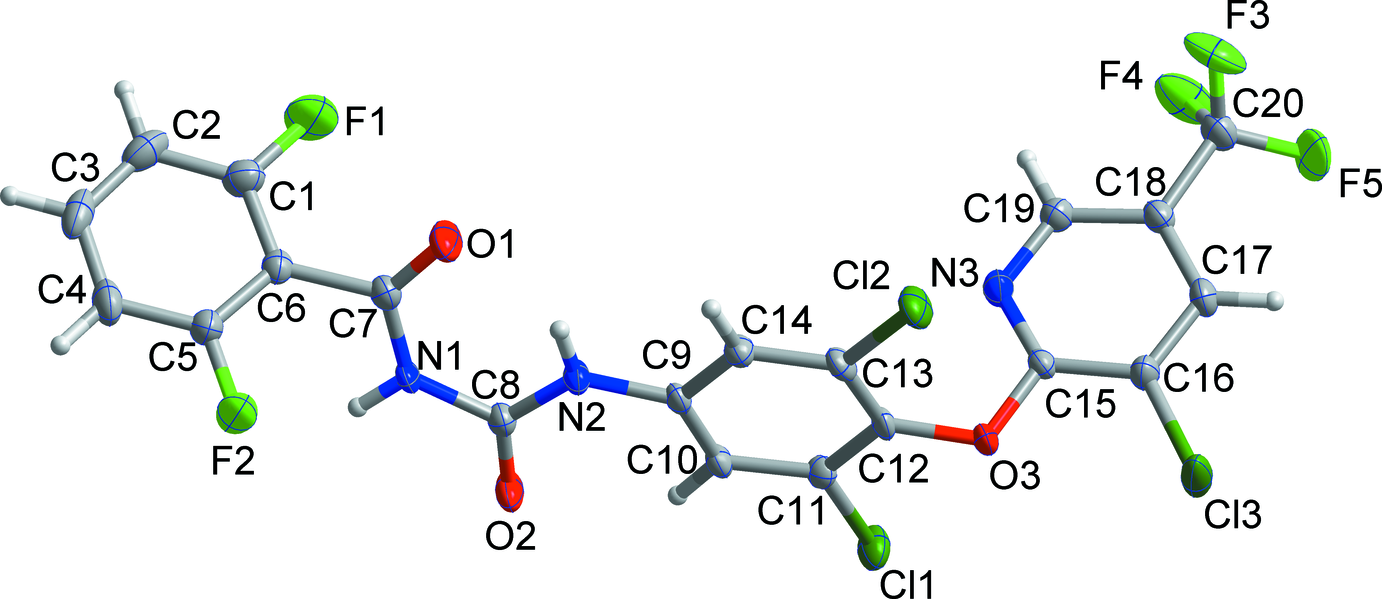

Supplement: Supplementary file 4 [file e-71-00o55-fig1.tif]

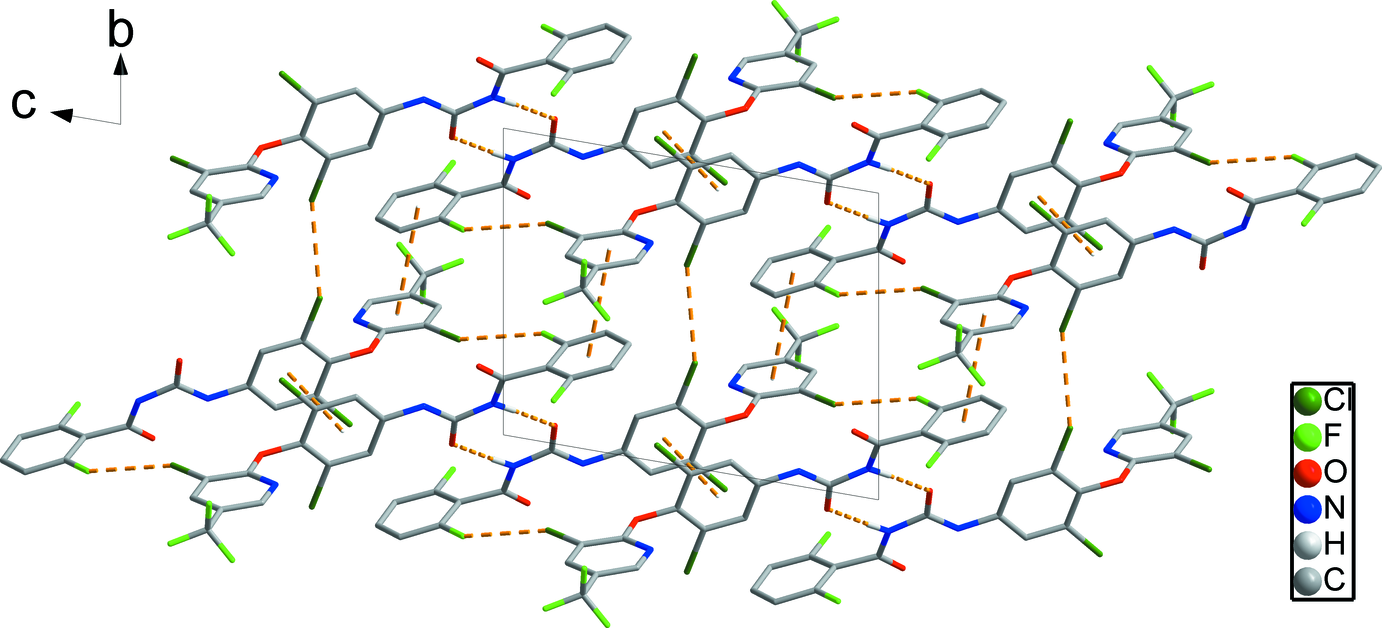

Supplement: Supplementary file 5 [file e-71-00o55-fig2.tif]
